# MYST1/KAT8 contributes to tumor progression by activating EGFR signaling in glioblastoma cells

**DOI:** 10.1002/cam4.2639

**Published:** 2019-11-05

**Authors:** Zhen Dong, Jiahua Zou, Jifu Li, Yi Pang, Yudong Liu, Chaowei Deng, Fei Chen, Hongjuan Cui

**Affiliations:** ^1^ State Key Laboratory of Silkworm Genome Biology Institute of Sericulture and Systems Biology Southwest University Chongqing China; ^2^ Cancer Center, Medical Research Institute Southwest University Chongqing China; ^3^ Engineering Research Center for Cancer Biomedical and Translational Medicine Southwest University Chongqing China; ^4^ Chongqing Engineering and Technology Research Center for Silk Biomaterials and Regenerative Medicine Southwest University Chongqing China; ^5^ College of Biotechnology Southwest University Chongqing China; ^6^ Chongqing Engineering Research Center of Antitumor Natural Drugs Chongqing Three Gorges Medical College Chongqing China; ^7^ Department of Pharmaceutical Sciences Eugene Applebaum College of Pharmacy and Health Sciences Wayne State University Detroit MI USA

**Keywords:** EGFR, glioblastoma, histone acetylation, KAT8, MYST1

## Abstract

With short survival time, glioblastoma (GBM) is the most malignant tumor in the central nervous system. Recently, epigenetic enzymes play essential roles in the regulation of tumorigenesis and cancer development of GBM. However, little is known about MYST1/KAT8/MOF, a histone acetylation enzyme, in GBM. The present study shows that MYST1 promotes GBM progression through activating epidermal growth factor receptor (EGFR) signaling. MYST1 expression was increased in GBM and was negatively correlated with prognosis in patients with glioma and GBM. Knockdown of MYST1 reduced cell proliferation and BrdU incorporation in LN229, U87, and A172 GBM cells. Besides, MYST1 downregulation also induced cell cycle arrest at G2M phase, as well as the reduced expression of CDK1, Cyclin A, Cyclin B1, and increased expression of p21^CIP1/Waf1^. Meanwhile, Self‐renewal capability in vitro and tumorigenecity in vivo were also impaired after MYST1 knockdown. Importantly, MYST1 expression was lowly expressed in mesenchymal subtype of GBM and was positively correlated with EGFR expression in a cohort from The Cancer Genome Atlas. Western blot subsequently confirmed that phosphorylation and activation of p‐Try1068 of EGFR, p‐Ser473 of AKT and p‐Thr202/Tyr204 of Erk1/2 were also decreased by MYST1 knockdown. Consistent with the results above, overexpression of MYST1 promoted GBM growth and activated EGFR signaling in vitro and in vivo. In addition, erlotinib, a US Food and Drug Administration approved cancer drug which targets EGFR, was able to rescue MYST1‐promoted cell proliferation and EGFR signaling pathway. Furthermore, the transcription of EGF, an EFGR ligand, was shown to be positively regulated by MYST1 possibly via H4K16 acetylation. Our findings elucidate MYST1 as a tumor promoter in GBM and an EGFR activator, and may be a potential drug target for GBM treatment.

## Graphical abstract

MYST1 is highly expressed in glioblastoma (GBM) and is negatively correlated with overall survival of patients with glioma and GBM, suggesting that its high expression predicts poor prognosis. These evidences indicate that MYST1 may play an important role in the development of GBM.

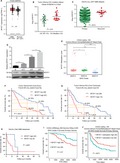

## 1 INTRODUCTION

Glioblastoma (GBM) is the most common and lethal malignant tumors in the central nervous system. It features strong aggressiveness, which makes the cells easily invade into the surrounding normal brain tissue.^1^ However, surgical clinical treatment can not completely remove the tumor tissue after surgery, because the remaining tumor cells may be stimulated to proliferate and invade into normal brain tissue to form a metastasized tumor.^2^ Although neurosurgery technology has greatly improved as well as chemotherapy, radiotherapy, and combined therapy with the application of biological treatment, the survival time of GBM patients is only 12‐15 months.^3^, ^4^ Therefore, it is urgent to explore the nature of GBM and the molecular mechanisms underlying for a better understanding of its malignancy, so as to develop a new method to cure GBM.

Epigenetic regulator plays important roles in GBM progression.^5^, ^6^, ^7^ MYST1, also termed as KAT8, MOZ, YBF2, SAS2, MOF, or TIP60 protein 1, is a kind of histone acetylation enzyme belonging to the MYST family and contains a chromodomain, a zinc finger motif, and a HAT domain.^8^, ^9^ As a catalytic subunit, MYST1 forms two distinct multiprotein complexes, MSL and NSL, which acetylates H4K16 as well as H4K5 and H4K8.^10^ MYST1 has been demonstrated to play important roles in embryonic formation and development, chromatin assembly, transcription activation, cellular apoptosis, double‐strand break repair, and cellular stress response.^11^, ^12^, ^13^, ^14^, ^15^, ^16^

Interestingly, MYST1 also showed capacities in the development of tumors.^10^ MYST1 is downregulated in medulloblastoma,^17^ primary breast carcinoma,^17^ ovarian epithelial cancer,^18^ colorectal carcinoma, gastric cancer, and renal cell carcinoma,^19^, ^20^ while upregulated in non‐small cell lung cancer (NSCLC).^21^ MYST1 acetylates Nrf2 at K588 and retains it in the nucleus, thereby regulating genes associate with anti‐oxidative and regulates anti‐drug responses in human NSCLC.^21^ MYST1 acetylates the histone demethylase LSD1 to impede its binding to epithelial gene promoters and histone demethylation, thus suppresses epithelial‐mesenchymal transition and tumor invasion.^22^ Besides, MYST1 is recruited by G9a‐dimethylated ERαK235 to the ERα target gene promoters to acetylate H4K16, thus promotes gene transcription in breast cancer.^16^ However, the correlation of MYST1 with the GBM has never been explored.

In this study, we showed that MYST1 is overexpressed in GBM and predicts a poor prognosis. Besides, we found that inhibition of MYST1 impedes epidermal growth factor receptor (EGFR) signaling and induces cell cycle arrest at G2M phase.

## 2 MATERIALS AND METHODS

### 2.1 Cell culture

Human GBM cell lines were maintained in Dulbecco's modified Eagle's medium (DMEM, Life Technologies; LN229, A172 cell line, ATCC) or Dulbecco's modified Eagle's medium: Nutrient Mixture F‐12 (DMEM/F‐12, Life Technologies; U87, U251 and U118 cell lines, ATCC) supplemented with 10% fetal bovine serum (FBS; Invitrogen) and 1% Penicillin‐Streptomycin (P/S, Invitrogen). HEB gliocytes (the Type Culture Collection of the Chinese Academy of Sciences) was also cultured in DMEM with 10% FBS and 1% P/S. Primary glioma cells was obtained using the glioma tissues provided by the Department of Neurosurgery, Daping Hospital, the Third Affiliated Hospital of Army Medical University. These cells were also cultured in DMEM with 10% FBS and 1% P/S. 293FT cell line (ATCC) were maintained in DMEM (DMEM, Life Technologies) supplemented with 10% FBS, 1% P/S, 1% MEM Non‐Essential Amino Acids Solution (Invitrogen), 2 mmol/L l‐Glutamine (Invitrogen) and 1 mmol/L Sodium Pyruvate (Invitrogen).

### 2.2 Reagents

Thymidine analog 5‐bromo‐2‐deoxyuridine (BrdU, B5002), Dimethyl sulfoxide (DMSO, D2650), and 3‐(4)‐2,5‐diphenyl‐2‐*H*‐tetrazolium bromide (MTT, M2128) were all obtained from Sigma‐Aldrich. Erlotinib (HY‐50896) was purchased from MedChemExpress.

### 2.3 Vector construction, transfection and infection

The RNAi target sites were designed and synthesized (the Beijing Genomics Institute, BGI) and were cloned into a lentiviral pLKO.1 vector. The sequences were listed as below: shMTST1‐F: CCGGGCAAGATCACTCGCAACCAAACTCGAGTTTGGTTGCGAGTGATCTTGCTTTTTG; shMYST1‐R: AATTCAAAAAGCAAGATCACTCGCAACCAAACTCGAGTTTGGTTGCGAGTGATCTTGC. Human full‐length MYST1 (NCBI Reference Sequence: NM_032188) cDNA was obtained using PCR and was cloned into lentiviral pCDH‐CMV‐MCS‐EF1‐copGFP vector. The primers used as below: MYST1‐F‐XbaI‐*Flag*: TGCTCTAGAATG*GATTACAAGGATGACGACGATAAG*GCGGCACAGGGAGCTGCTGCGG; MYST1‐R‐BamHI‐Flag: CGCGGATCCTCA*CTTATCGTCGTCATCCTTGTAATC*CTTCTTGGAGAGCTTGACTTGC. Letivirural vector construction, transfection, and infection were employed as described previously.^23^

### 2.4 Quantitative RT‐PCR

Total RNA was extracted using RNAiso Plus (Takara, Dalian, China) according to manufacturer's protocols and real‐time qPCR was conducted as reported previously.^24^ Expression analysis was performed by virtue of the ΔΔ*C_t_* method with glyceraldehyde‐3‐phosphate dehydrogenase (GAPDH) as a control. The primers were listed as below: GAPDH‐F: AACGGATTTGGTCGTATTGGG, GAPDH‐R: CCTGGAAGATGGTGATGGGAT, MYST1‐F: GGTGGAGATCGGAGAAACGTA, MYST1‐R: CAGCATCCTTCACTGTCTTGGT, EGFR‐F: AAAGTTAAAATTCCCGTCGCTATCAAG, EGFR‐R: TCACGTAGGCTTCATCGAGGATTTC. EGF‐F: TGGTGATGGGAGGATGACTTG, EGF‐R: GGCCAGTGACTCAGCAGAAA. Primers were designed according to a previous study.^25^

### 2.5 Western blot

Western blot was performed as described previously.^24^ The antibodies used were listed as below: MYST1 (Rabbit mAb #46862, Cell Signaling Technology, CST), CDK1 (Cdc2, Rabbit mAb #77055, CST), Cyclin A (Mouse IgG2a #sc‐271682, Santa Cruz Biotechnology, SCB), Cyclin B1 (Mouse IgG1 # sc‐70898, SCB), p21 (Mouse IgG2a #sc‐71811), p‐Tyr1068‐EGFR (Rabbit mAb #3777, CST), EGFR (Rabbit mAb #E021073‐1, EnoGene), pSer473‐AKT (Rabbit mAb #4060, CST), AKT (Rabbit IgG #sc‐8312, SCB), pThr202/Tyr204‐Erk1/2 (Rabbit mAb #4370, CST), Erk1/2 (Rabbit mAb #9102, CST), H4K16ac (Rabbit IgG #ab109463, Abcam), Histone H4 (Rabbit IgG #16047‐1‐AP, Proteintech) and GAPDH (Mouse mAb # AF0006, Beyotime).

### 2.6 BrdU assay

The BrdU assay was performed according to previous description.^26^ BrdU antibody (Rat mAb # ab6326, Abcam, Shanghai, China) and Goat anti‐Rabbit IgG (H+L) Cross‐Adsorbed ReadyProbes™ Secondary Antibody, Alexa Fluor 594 (#R37117 Invitrogen, Thermo Fisher Scientific) were used in this experiment.

### 2.7 MTT assay and trypan blue assay

MTT assay were conducted as described previously.^27^ Typan blue assay was described briefly as below: 2 × 10^5^ Cells were seeded on six‐well plates and after indicated time cells were trypsinized and responded adequately with an equal volume of trypan blue (0.4%), then living cells without blue staining was calculated under a microscope.

### 2.8 Cell cycle

Detection of cell cycle was performed according to previous report.^24^

### 2.9 Soft agar assay

Colony formation ability was determined by soft agar assay on LN229 and U87 cells by virtue of the method provided previously.^28^

### 2.10 Tumor xenografts

The female mice (BALA/c‐nu, Beijing Huafukang Bioscience Co. Inc, China) with 4‐weeks‐old were purchased and housed in a specific‐pathogen‐free room. LN229 and U87 cells (1 × 10^6^) with gene alterations in 100 μL DMEM without FBS were subcutaneously injected into both flanks of the mice. Every group contains 4‐6 mice. Tumor growth was measured by caliper measurement every four days after tumor plumped, and tumor volume was calculated with the formula (volume = tumor length × width^2^ × *π*/6). After 48‐52 days, tumors were removed and weighed. All animal experiments were conducted according to the Declaration of Helsinki and was pre‐approved and supervised by the Institutional Animal Care and Use Committees of the Southwest University and Experimental Animal Care and Use Committees of the Institute of Sericulture and Systems Biology.

### 2.11 Immunocytochemistry

Immunocytochemistry was performed as described previously.^26^ MYST1 (Rabbit mAb # ab200660, Abcam) and Ki67 (#550609, BD pharmingen) antibodies, Histostain™—SP Kits (#SPN‐9002, ZSGB‐BIO) were used in this experiment.

### 2.12 Enzyme‐linked immuno sorbent assay

Cells (2 × 10^4^ per well) was plated in a 96‐well plate and Enzyme‐linked immuno sorbent assay (ELISA) was performed to detected the EGF levels in the medium after cultured for 48 hours using the Human EGF Quantikine ELISA Kit (#DEG00, R&D Systems) according to manufacture's protocols.

### 2.13 Clinical data, ChIP‐seq data, and Statistical analysis

All data analysis was performed using a software GraphPad Prism 8 (https://www.graphpad.com/). Unpaired two‐tailed Student's *t* test was used for statistical analysis between two groups. *P* < .05 was considered statistically significant and was marked with * in the figures. *P* < .01 was marked with **. *P* < .001 was marked with ***. The Cancer Genome Atlas (TCGA) data of MYST1 mRNA expression in GBM and normal tissues were obtained and analyzed in the Gene Expressing Profiling Interactive Analysis (GEPIA, http://gepia.cancer-pku.cn/index.html).^29^ MYST1 mRNA expression data from the database termed Tumor Glioma (CIC mutation status) Gleize‐30‐MAS5.0‐u133p2, prognosis data from databases termed Tumor Glioma‐IGS (Core‐Transcript) French‐95‐rma_sketch‐huex10t and Tumor Glioma‐IGS (Core‐Exon) French‐95‐rma_sketch‐huex10p, MYST1 expression data in different subtypes and data of MYST1 coexpression with EGFR, AKT1, and MAPK3 from Tumor GBM TCGA‐540‐MAS5.0‐u133a were downloaded from the R2 platform (https://hgserver1.amc.nl/). Log‐rank (Mentel‐Cox) test was conducted for the significance in survival analysis. Scan cutoff modus was used to get the most significant expression cutoff in the Kaplan Meier module for survival analysis. The data of MYST1 expression data in different subtypes from TCGA—GBM Affymetrix HT HG U133A were obtained from Betastasis (http://www.betastasis.com/). The data of MYST1 mRNA expression in the primary and recurrent GBM and survival, as well as the data of correlations of MYST1 mRNA level and EGF (epidermal growth factor), TGFA (transforming growth factor alpha), AREG (amphiregulin), and EREG (epiregulin) mRNA levels were acquired from GlioVis, data visualization tools for brain tumor datasets (http://gliovis.bioinfo.cnio.es/).^30^ The data of MYST1 gene methylation level and its correlation with prognosis in different WHO grades of glioma was obtained from the Chinese Glioma Genome Atlas (CGGA, http://www.cgga.org.cn/). One‐way analysis of variance (ANOVA) multiple comparison was performed for MYST1 gene expression pattern in different glioma subgroups. ChIP‐seq data of H4K16ac in human fibroblast IMR90 cells using chip antibodies H4K16ac (Millipore 07‐329, GSM1358821_H4K16ac.Prolif.R1) or H4K16ac (Abcam ab109463, GSM1358822_H4K16ac.Prolif.R2)^31^ were downloaded from GEO (https://www.ncbi.nlm.nih.gov/geo/) and analyzed using the software Integrative genomics viewer version 2.6.3.^32^, ^33^, ^34^

## 3 RESULTS

### 3.1 MYST1 is correlated with the prognosis of patients with glioma

**Figure 1:**
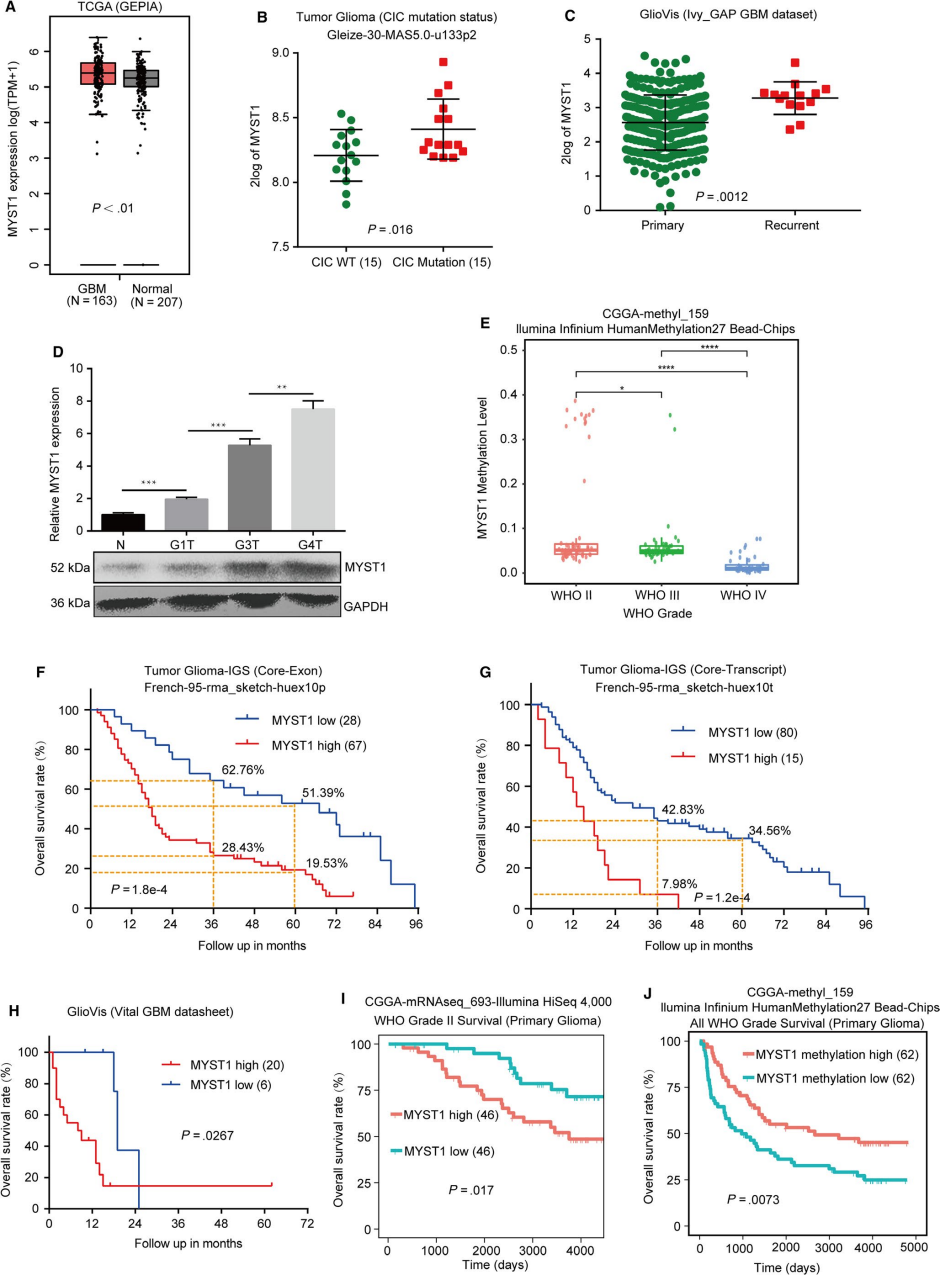
MYST1 is correlated with the prognosis of patients with glioma. A, The analysis of TCGA data of MYST1 mRNA expression in GBM and normal tissues were obtained and analyzed in the GEPIA. B, The analysis of MYST1 mRNA expression in gliomas with CIC mutation from the database termed Tumor Glioma (CIC mutation status) Gleize‐30‐MAS5.0‐u133p2. C, The analysis of MYST1 mRNA expression in the primary and recurrent GBM in the data acquired from the GlioVis. D, The relative mRNA level and protein level of MYST1 in normal HEB gliocytes (N) and primary cells derived from different grades of gliomas. G1T, grade I gliomas; G3T, grade III gliomas; G4T, grade IV gliomas. E, MYST1 gene methylation levels of different WHO grades of gliomas in the CGGA methyl‐159 datasheet. F and G, Kaplan‐Meier (KM) analysis and Log‐rank test demonstrated that the elevated MYST1 protein expression in glioma is correlated with shorter overall survival in two connected databases titled with Tumor Glioma‐IGS (Core‐Transcript) French‐95‐rma_sketch‐huex10t and Tumor Glioma‐IGS (Core‐Exon) French‐95‐rma_sketch‐huex10p from the R2 microarray analysis and visualization platform. H, KM analysis and Log‐rank test of MYST1 expression data in different subtypes from Vital GBM datasheet downloaded from GlioVis. I, KM analysis and Log‐rank test of MYST1 expression data in WHO grade II gliomas in the datasheet downloaded from the CGGA. J, KM analysis and Log‐rank test of MYST1 methylation level in gliomas in the datasheet downloaded from the CGGA. Experimental data were used as mean ± SD, n ≥ 3, significant difference was tested by Student's *t* test. *P < .05, **P < .01, ***P < .001, ****P < .0001. *P* < .05 was considered as statistically significant. GBM, glioblastoma; GEPIA, Gene Expressing Profiling Interactive Analysis

To reveal the function of MYST1, we firstly explored its expression in GBM and normal tissues in the TCGA database downloaded from the GEPIA. The result showed MYST1 mRNA expression was highly expressed in GBM, compared with that of normal brain tissues (Figure 1A). Since CIC mutation predicted poor outcome of glioma patients,^35^, ^36^ we analyzed MYST1 expression in gliomas with CIC wildtype or mutation in the database termed Tumor Glioma (CIC mutation status) Gleize‐30‐MAS5.0‐u133p2. The result showed that MYST1 expression was higher in gliomas with CIC mutation than that of gliomas with CIC wildtype (Figure 1B). Besides, MYST1 also increased its expression in recurrent GBM (Figure 1C). Importantly, qRT‐PCR and Western blot showed that MYST1 mRNA and protein expression were increased in higher grades of gliomas (Figure 1D). Since methylation of DNA affects the expression of genes, we further found that MYST1 methylation level were lower in WHO higher grade of gliomas than that of the lower grade in the CGGA datasheet (Figure 1E). Then we analyzed the relationship of MYST1 expression and the prognosis of glioma patients in two databases titled Tumor Glioma‐IGS (Core‐Transcript) French‐95‐rma_sketch‐huex10t and Tumor Glioma‐IGS (Core‐Exon) French‐95‐rma_sketch‐huex10p by virtue of Kaplan‐Meier (KM) analysis. The result showed that MYST1 expression was negatively correlated with the overall survival (OS) rate in 95 patients with glioma in two databases (*P*‐value = 1.2e‐4 and 1.8e‐4, respectively, Figure 1F,G). Besides, 3 or 5‐year OS rate is also higher in patients with elevated MYST1 expression than that with low MYST1 expression (Figure 1F,G). In the database of GBM termed Vital GBM datasheet from GlioVis, MYST1 high expression also predicts poor prognosis (Figure 1H). In a WHO grade II cohort in the CGGA database, we also found that MYST1 expression was negatively associated with the prognosis (Figure 1I). Besides which, MYST1 gene methylation level was positively correlated with prognosis of patients with gliomas, according to the data from a gene methylation database of gliomas in the CGGA (Figure 1J). These results showed that MYST1 is highly expressed in GBM and predicts poor prognosis of GBM patients.

### 3.2 MYST1 silencing inhibits cell proliferation in GBM cells

**Figure 2:**
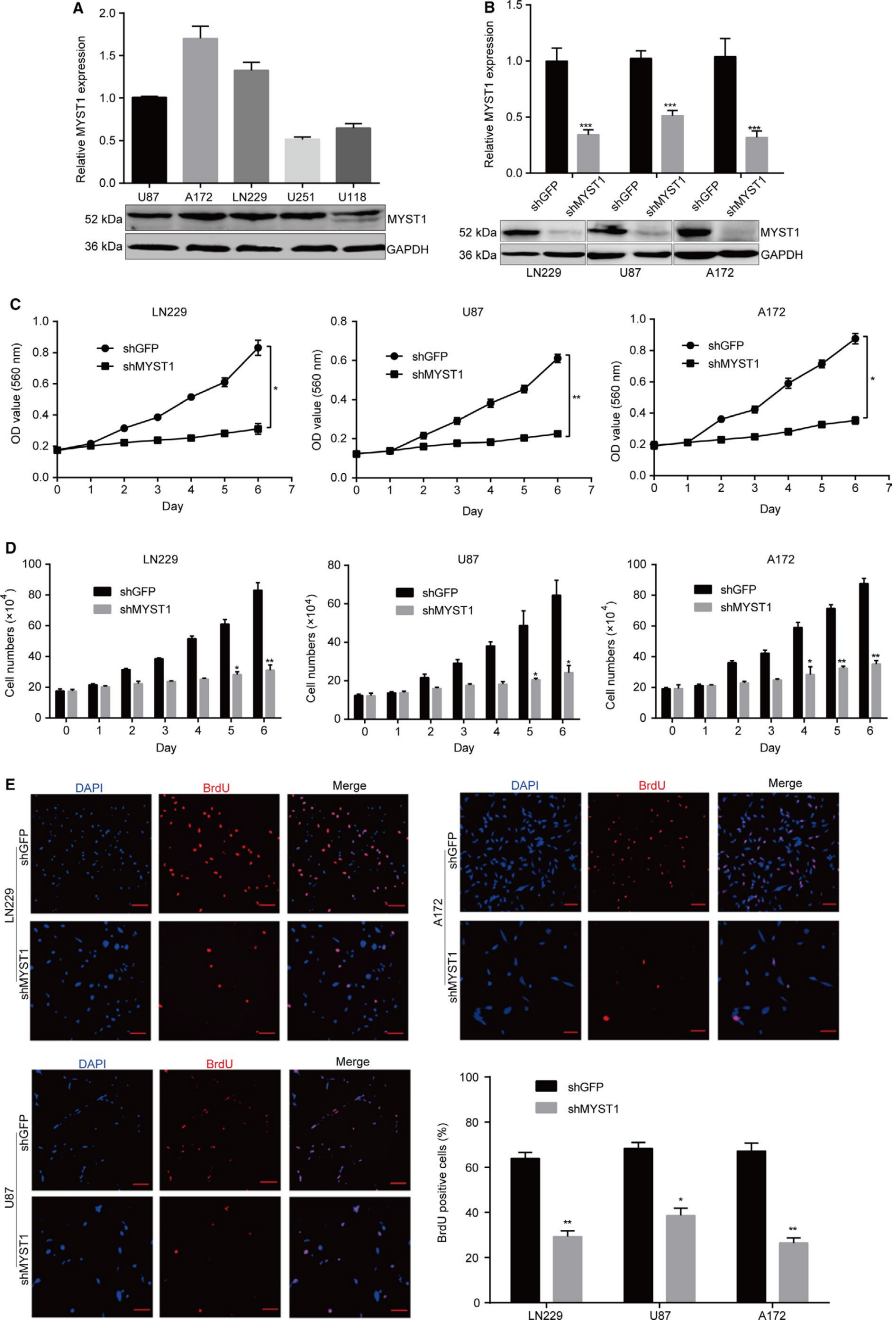
MYST1 silencing inhibits cell proliferation in GBM cells. A, Quantitative real‐time PCR (qRT‐PCR) and western blot analysis of MYST1 mRNA and protein expression in several GBM cell lines. B, qRT‐PCR and Western blot analysis of MYST1 mRNA and protein expression in LN229, U87 and A172 cells after MYST1/GFP silencing. C, MTT assay reveals significant cell proliferation inhibition induced by MYST1 silencing in LN229, U87 and A172 cells, compared to that of the GFP silencing group (n = 3). D, Cell counting by trypan blue assay reveals significant cell proliferation inhibition induced by MYST1 silencing in LN229, U87 and A172 cells, compared to that of the GFP silencing group (n = 3). E, BrdU‐positive cells in LN229, U87 and A172 after MYST1/GFP silencing. All data were used as mean ± SD, n ≥ 3, significant difference was tested by Student's *t* test. *P < .05, **P < .01, ***P < .001. *P* < .05 was considered as statistically significant. GBM, glioblastoma; GFP, green fluorescent protein

Next, we analyzed MYST1 mRNA and protein expression in several GBM cell lines including U87, A172, LN229, U251, and U118. The results showed that MYST1 was commonly expressed in these cell lines (Figure 2A). Since MYST1 expression was high in LN229, U87, and A172 cells, we used them in our subsequent studies. To further elucidate the function of MYST1 in GBM cells, we silenced MYST1 mRNA and protein expression in LN229, U87, and A172 cells using lentivirus‐mediated shRNA stable transfection (Figure 2B). Significantly, MYST1 silencing decreased cell viability and cell number in three cell lines, compared with an inactive green fluorescent protein (GFP) silencing (Figure 2C,D). Besides, MYST1 silencing also reduced BrdU incorporation in these cells (Figure 2E). These results indicated that MYST1 silencing inhibits cell proliferation in GBM cells.

### 3.3 MYST1 silencing induces cell cycle arrest at G2/M phase

**Figure 3:**
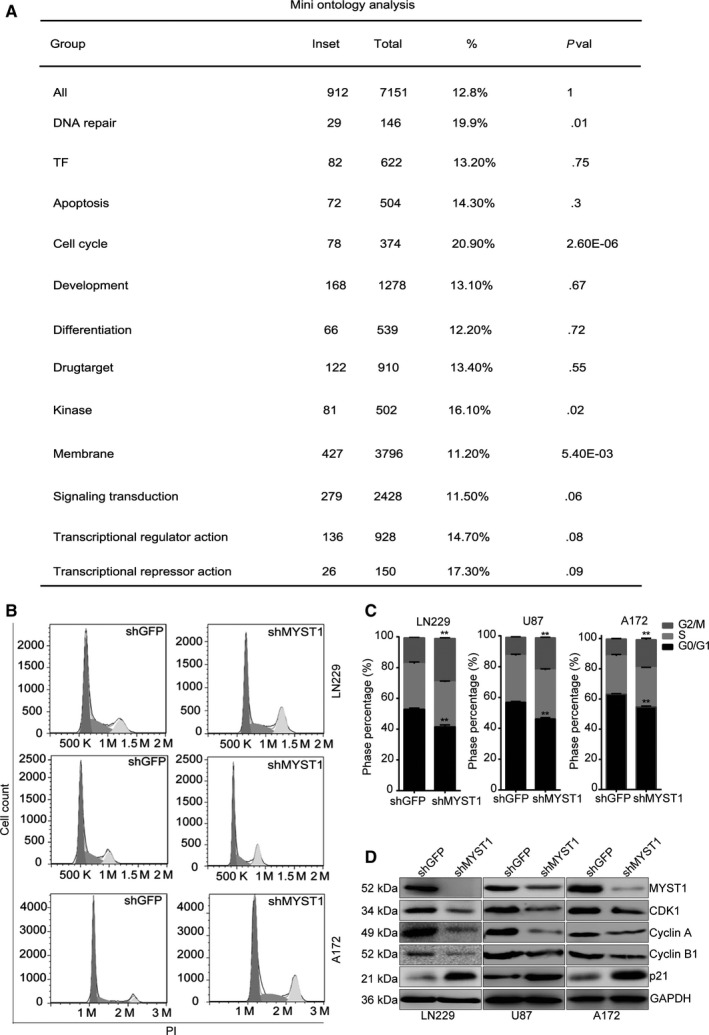
MYST1 silencing induces cell cycle arrest at G2/M phase. A, Mini ontology analysis of genes correlated with MYST1 expression in a database titled Tumor Glioma‐IGS (Core‐Exon) French‐95‐rma_sketch‐huex10p from the R2 microarray analysis and visualization platform. B and C, Cell cycle analysis of the propidium iodide (PI) staining in LN229, U87 and A172 cells after MYST1/GFP silencing. D, Western blots of cell cycle‐related proteins expression in LN229, U87 and A172 cells after MYST1/GFP silencing. All data were used as mean ± SD, n ≥ 3, significant difference was tested by Student's *t* test. **P < .01. *P* < .05 was considered as statistically significant. GFP, green fluorescent protein

To further explain these results, we analyzed genes correlated with MYST1 expression in a cohort titled Tumor Glioma‐IGS (Core‐Exon) French‐95‐rma_sketch‐huex10p. The mini ontology analysis showed that genes participated into biological process including cell cycle (*P*‐value = 2.6e‐6), membrane (*P*‐value = 5.4e‐3), DNA repair (*P*‐value = .01), and kinase (*P*‐value = 0.02) have significant correlations with the expression of MYST1 in glioma (Figure 3A). Since cell cycle is the most significant biological process, we focus our subsequent study on this topic. Flow cytometry analysis of propidium iodide staining showed that MYST1 silencing induced cell cycle arrest at G2M phase in LN229, U87 and A172 cells, compared with an inactive GFP silencing (Figure 3B,C). Besides, we found that the expression of cell cycle‐related proteins such as CDK1, Cyclin A, and CyclinB1 were significantly reduced after MYST1 knockdown, while the expression of CDK inhibitor p21 was significantly upregulated after MYST1 knockdown (Figure 3D). These results showed that MYST1 silencing probably inhibits cell proliferation via regulation of cell cycle progression.

### 3.4 MYST1 silencing suppresses tumorigenecity of GBM cells

**Figure 4:**
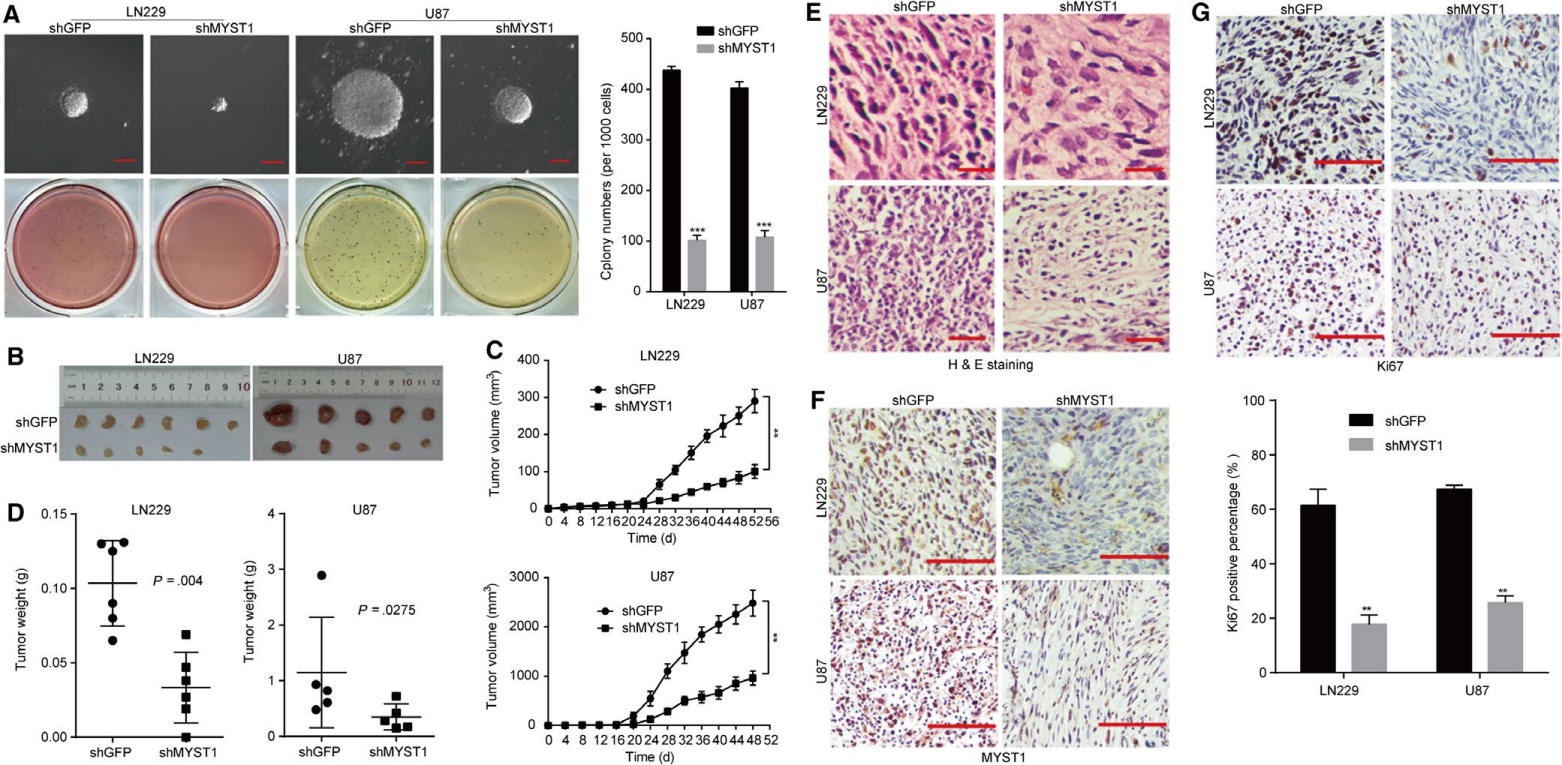
MYST1 silencing suppresses tumorigenecity of GBM cells. A, Soft agar assay was performed to test the colony‐formation ability of LN229, U87 and A172 cells after MYST1/GFP silencing. B‐D, The effect of MYST1/GFP silencing on tumorigenicity in BALB/c‐nu nude mice. LN229 and U87 cells with MYST1/GFP silencing were injected subcutaneously into the nude mice (n = 6 or 5). Tumor burden was monitored every 4 d by a digital caliper. Tumor size was calculated as the formula: length × width^2^ × *π*/6. After the experiment, mice were sacrificed and the tumor was taken out and weighted. E, H&E staining and IHC of Ki67 and MYST1 in indicated tumors obtained from the nude mice. All data were used as mean ± SD, n ≥ 3, significant difference was tested by Student's *t* test. **P < .01, ***P < .001, *P* < .05 was considered as statistically significant. GBM, glioblastoma; GFP, green fluorescent protein; H&E, Hematoxylin‐eosin

Furthermore, we confirmed the effect of MYST1 silencing in GBM cells in vivo. Before that, we firstly tested the self‐renewal capacity of cells after MYST1 silencing by virtue of soft agar experiment. Significantly, MYST1 silencing reduced the size and number of colony formation in LN229 and U87 cells, compared with GFP silencing (Figure 4A). Based on the results obtained above, we tried to explore the role of MYST1 in tumor progression in vivo by injecting LN229‐shGFP, LN229‐shMYST1, U87‐shGFP, and U87‐shMYST1 cells subcutaneously in the flanks of the BALB/c‐nu mice. We observed that the tumor volumes and weights of all MYST1‐silenced tumors in the nude mice were significantly smaller than the control groups after 48‐52 days, respectively (Figure 4B‐D). Hematoxylin‐eosin staining revealed that tumor malignancy (nucleocytoplasmic ratio) was decreased after MYST1 knockdown (Figure 4D). Immunostaining (IHC) analysis further confirmed the downregulation of MYST1 and Ki67 protein in MYST1‐silenced cells (Figure 4F,G). Taken together, these results suggested that MTST1 is essential for the tumorigenecity of GBM cells in vivo.

### 3.5 MYST1 silencing downregulates EGFR signaling pathway

**Figure 5:**
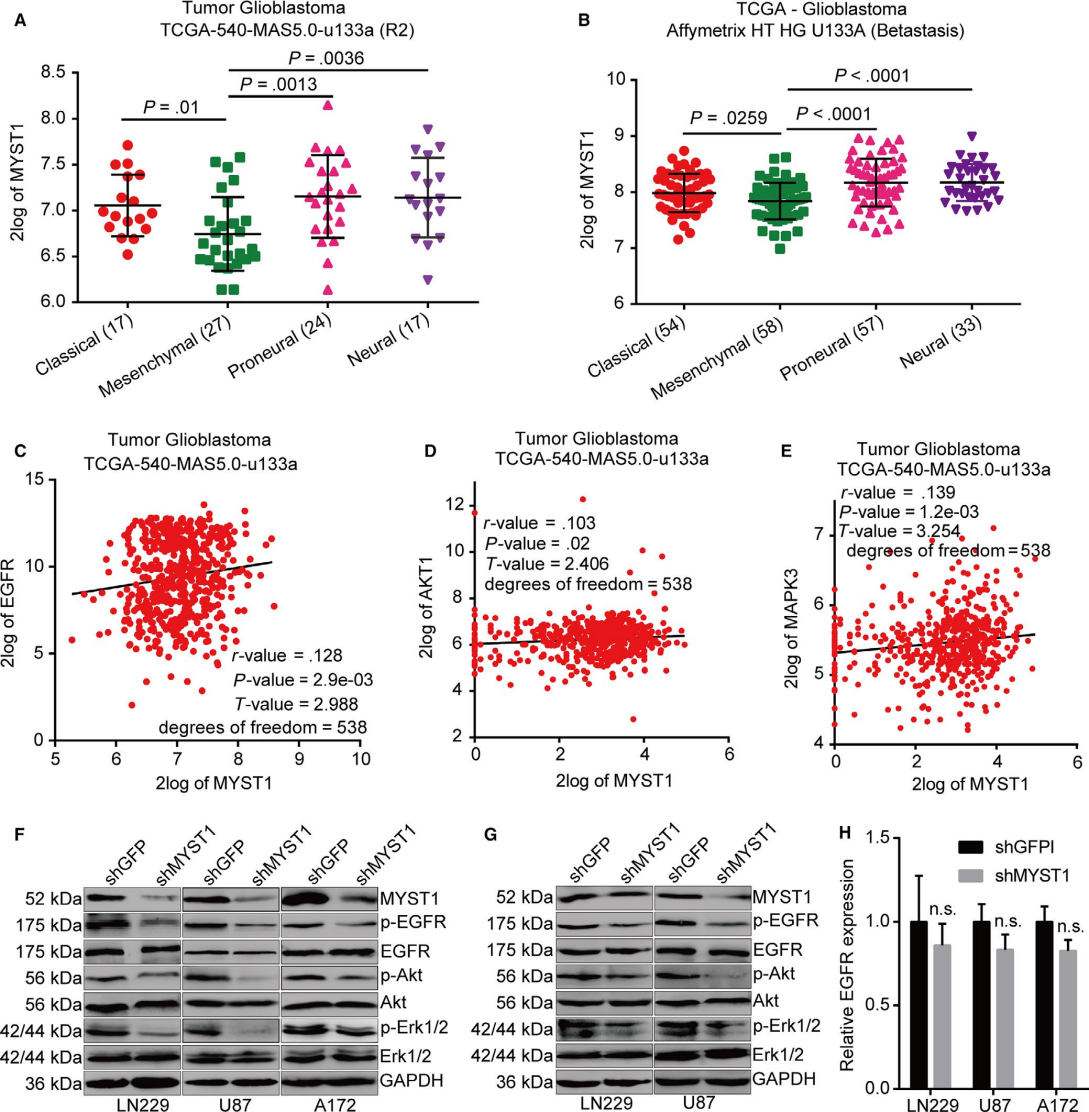
MYST1 silencing downregulates EGFR signaling pathway. A, The analysis of MYST1 expression in different subtypes of GBM from a TCGA cohort titled Tumor Glioblastoma TCGA‐540‐MAS5.0‐u133a in the R2 microarray analysis and visualization platform. B, The analysis of MYST1 expression in different subtypes of GBM from TCGA—Glioblastoma Affymetrix HT HG U133A were obtained from Betastasis (http://www.betastasis.com/). C‐E, The correlations of two genes analysis in in a TCGA cohort titled Tumor Glioblastoma TCGA‐540‐MAS5.0‐u133a from the R2 microarray analysis and visualization platform. F, Western blots of proteins expression in EGFR signaling pathways in LN229, U87 and A172 cells after MYST1/GFP silencing. G, Western blots of proteins expression in EGFR signaling pathways in indicated tumors obtained from the nude mice. H, qRT‐PCR analysis of EGFR expression after MYST1 knockdown. Experimental data were used as mean ± SD, n ≥ 3, significant difference was tested by Student's *t* test. n.s.= no sense. *P* < .05 was considered as statistically significant. EGFR, epidermal growth factor receptor; GBM, glioblastoma; GFP, green fluorescent protein

Genetic alterations in GBM have been previously identified, with frequent amplification of some receptor‐tyrosine kinases such as EGFR, platelet‐derived growth factor receptor alpha (PDGFRA), and Met Proto‐Oncogene (MET).^37^, ^38^, ^39^ Among them, EGFR is found to be amplificated in about 57% GBM and is a clinical target for GBM harboring EGFR activation.^38^ Besides, EGFR amplification is lower in mesenchymal subtype of GBM cases than other three types, such as classical, neural, and proneural subtypes.^40^ Interestingly, we found that MYST1 expression was also lower in mesenchymal subtype of GBM cases than other three types (Figure 5A,B). Importantly, we found that MYST1 expression was significantly correlated with the expression of EGFR and its downstream target, such as AKT1 and MAPK3 (ERK1) in a TCGA cohort titled Tumor GBM TCGA‐540‐MAS5.0‐u133a (Figure 5C‐E). Besides, the phosphorylation of Tyr1068 in EGFR, Ser473 in AKT and Thr202/Tyr204 in Erk1/2 were declined in three GBM cell lines after MYST1 silencing, compared with GFP silencing, indicating EGFR‐AKT‐ERK1/2 signaling pathway was downregulated by MYST1 knockdown (Figure 5F). Consistently, EGFR‐AKT‐ERK1/2 signaling pathway was downregulated in xenografts obtained above after MYST1 silencing, compared with GFP silencing (Figure 5G). However, EGFR mRNA expression remained unchanged after MYST1 silencing (Figure 5H). These results indicated that MYST1 might promote EGFR signaling pathway in GBM.

### 3.6 MYST1 overexpression promotes GBM progression and upregulates EGFR signaling pathway

**Figure 6:**
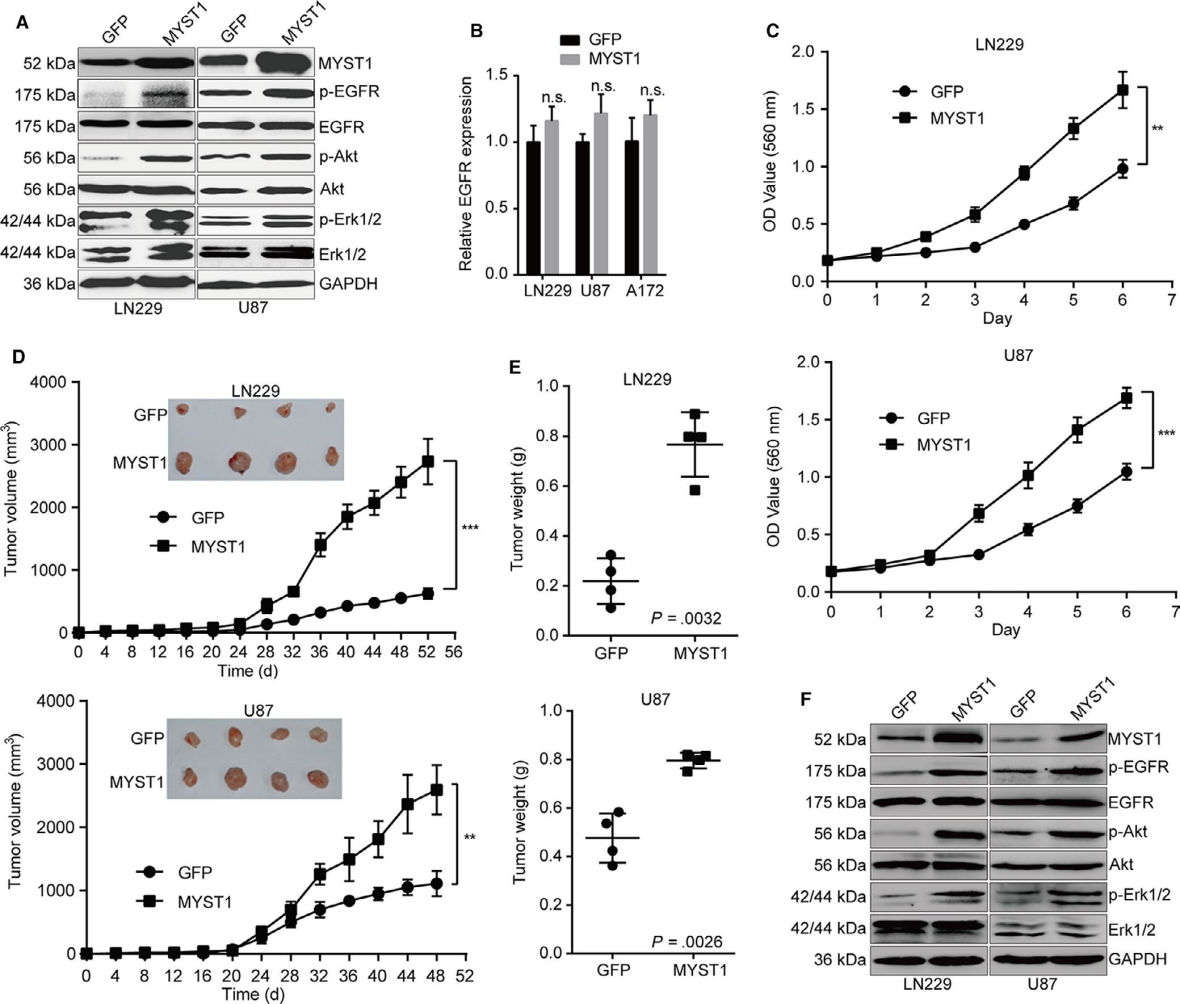
MYST1 overexpression promotes GBM progression and upregulates EGFR signaling pathway. A, Western blots of proteins expression in EGFR signaling pathways in LN229 and U87 cells after MYST1/GFP overexpression. B, qRT‐PCR analysis of EGFR mRNA expression after MYST1 overexpression. C, MTT assay reveals significant cell proliferation promotion induced by MYST1/GFP overexpression in LN229 and U87 cells (n = 3). D and E, The effect of MYST1/GFP silencing on tumorigenicity in BALB/c‐nu nude mice. LN229 and U87 cells with MYST1/GFP overexpression were injected subcutaneously into the nude mice (n = 4). Tumor burden was monitored every 4 d by a digital caliper. Tumor size was calculated as the formula: length × width^2^ × π/6. After the experiment, mice were sacrificed and the tumor was taken out and weighted. F, Western blots of proteins expression in EGFR signaling pathways in indicated tumors obtained from the nude mice. All data were used as mean ± SD, n ≥ 3, significant difference was tested by STUDENT's *t* test. **P < .01, ***P < .001. *P* < .05 was considered as statistically significant. EGFR, epidermal growth factor receptor; GBM, glioblastoma; GFP, green fluorescent protein

To further confirm the results above, we overexpressed MYST1 in LN229 and U87 cells (Figure 6A). The results showed that MYST1 overexpression promoted EGFR signaling pathway and cell proliferation in vitro using the MTT method, compared with GFP‐overexpressed groups (Figure 6A,C). However, EGFR mRNA expression remained unchanged after MYST1 overexpression (Figure 6B). Moreover, MYST1 overexpression also promoted tumor growth and EGFR activation in vivo (Figure 6D‐F). Taken together, these results confirmed the role of MYST1 in the progression of GBM.

### 3.7 Inhibition of EGFR by erlotinib recovered GBM progression induced by MYST1 overexpression

**Figure 7:**
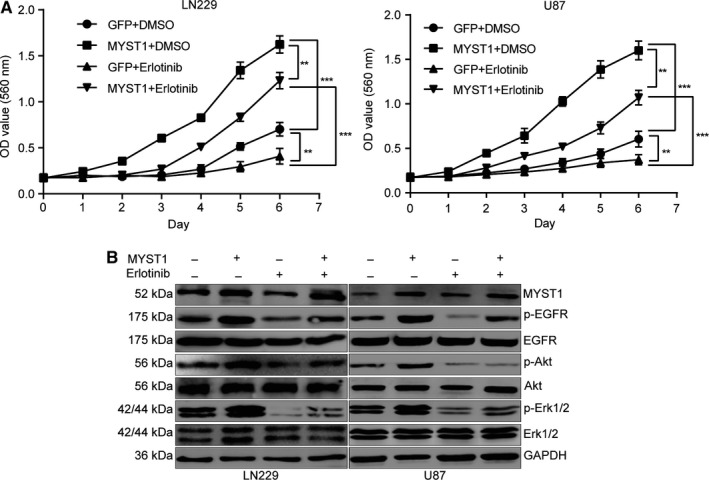
Inhibition of EGFR by erlotinib recovered GBM progression induced by MYST1 overexpression. A, MTT assay to detect cell proliferation in LN229 and U87 cells with MYST1/GFP overexpression after erlotinib (200 ng/mL)/DMSO treatment (n = 3). B, Western blots of proteins expression in EGFR signaling pathways in LN229 and U87 cells with MYST1/GFP overexpression after erlotinib (200 ng/mL)/DMSO treatment. All data were used as mean ± SD, n ≥ 3, significant difference was tested by Student's *t* test. **P < .01, ***P < .001. *P* < .05 was considered as statistically significant. EGFR, epidermal growth factor receptor; GBM, glioblastoma; GFP, green fluorescent protein

To further confirm that MYST1 promoted cell proliferation through EGFR activation, we used an EGFR inhibitor, erlotinib, which was used to treat NSCLC, pancreatic cancer and several other types of cancer. The results showed that MYST1‐promoted cell proliferation was blocked, at least partly, by erlotinib treatment (Figure 7A). Besides, MYST1‐promoted EGFR signaling activation was also diminished by erlotinib treatment (Figure 7B). These results indicated that MYST1 promoted cell proliferation through EGFR activation in GBM.

### 3.8 MYST1 regulates EGF expression possibly via H4K16 acetylation

**Figure 8:**
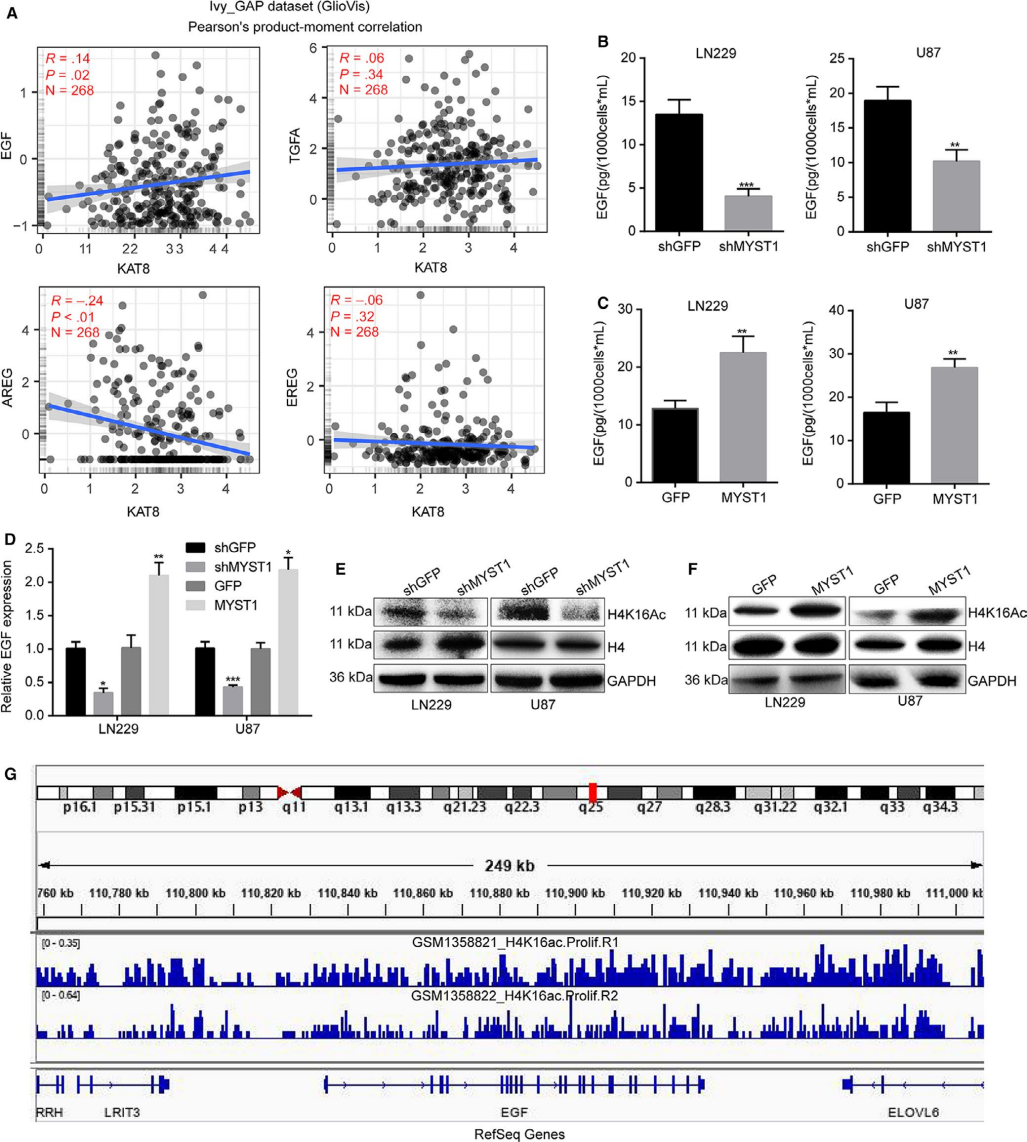
MYST1 regulates EGF expression possibly via H4K16 acetylation. A, The correlations of MYST1 mRNA level with the levels of several EGFR ligands including EGF, TGFA, AREG, and EREG in the datasheet from the GlioVis. B and C, ELISA assay was performed to detect the EGF protein levels in the medium of LN229 and U87 cells after MYST1 silencing or overexpression and cultured for 48 h within 10 mL medium. D, Relative mRNA levels of EGF in LN229 and U87 cells after MYST1 silencing or overexpression. E and F, Western blot was performed to detect the protein levels of H4K16 acetylation in LN229 and U87 cells after MYST1 silencing or overexpression. Histone H4 and GAPDH was used as controls. G, ChIP‐seq data of H4K16ac in human fibroblast IMR90 cells using chip antibodies H4K16ac (Millipore 07‐329, GSM1358821_H4K16ac.Prolif.R1) or H4K16ac (Abcam ab109463, GSM1358822_H4K16ac.Prolif.R2) was downloaded from GEO and analyzed by using the IGV 2.6.3 software. All data were used as mean ± SD, n ≥ 3, significant difference was tested by Student's *t* test. *P < .05, **P < .01, ***P < .001. *P* < .05 were considered as statistically significant. EGFR, epidermal growth factor receptor; ELISA, Enzyme‐linked immuno sorbent assay

Subsequently, we explored the mechanism of EGFR signaling activated by MYST1 in GBM. Since EGFR signaling can be activated by its ligands, such as EGF, TGFA, AREG, and EREG, we firstly analyzed the correlations of MYST1 mRNA level with the levels of these ligands in the datasheet from the GlioVis. The results showed that only EGF expression was positively correlated with MYST1 expression (Figure 8A). Then we performed ELISA assay to detect the EGF protein levels in the medium of LN229 and U87 cells after MYST1 silencing or overexpression. The results revealed that EGF protein level was decreased after MYST1 silencing, while was increased after MYST1 overexpression (Figure 8B,C). In consistent with the results above, mRNA levels of EGF in LN229 and U87 cells was also downregulated after MYST1 silencing and upregulated after MYST1 overexpression (Figure 8D). We suspected that H4K16 acetylation controlled by MYST1 might play a role in the expression of EGF. Western blot showed that levels of H4K16 acetylation in LN229 and U87 cells was decreased after MYST1 silencing and increased after MYST1 overexpression (Figure 8E,F). Importantly, ChIP‐seq data of H4K16ac in human fibroblast IMR90 cells using ChIP‐grade H4K16ac antibodies downloaded from GEO also showed that H4K16ac was enriched in the DNA sequence of EGF genes (Figure 8G). These results implied that MYST1 activates EGFR signaling possibly via epigenetically promoting the transcription of EGF.

## 4 DISCUSSION

Accumulating evidence demonstrated that MYST1 was correlated with the progression of various cancer cell types including medulloblastoma, breast cancer, ovarian cancer, colorectal carcinoma, gastric cancer, renal cell carcinoma, and NSCLC.^17^, ^18^, ^19^, ^20^, ^21^ Here we described the functional significance of MYST1 expression in GBM.

Glioblastoma is the most malignant tumor in the brain and has a very poor prognosis. Despite many efforts has been made to treat GBM, little effect is achieved on curing it.^41^ Therefore, it is urgent to make a better understanding about this disease and to develop a more effect therapeutic regimen. As a tyrosine kinase receptor of the ErbB family, EGFR is a 170 kDa glycoprotein with intracellular, extracellular, and transmembrane domains. Molecular analysis has revealed that about half of the GBMs have EGFR amplification, overexpression and/or EGFRvIII mutations.^42^, ^43^ EGFR confers a variety of malignant effects in GBM, including promotion of tumor growth, cell motility, and invasion, resistance to radiation and chemotherapy, maintenance of heterogeneity, etc.^44^, ^45^ EGFR can be activated through autophosphorylation of their intracellular domains, which subsequently activate^46^ the PI3K/Akt^46^ and MEK (MAPK‐ERK kinase) signaling pathways.^47^ Especially, the phosphorylation of Tyr1068 of EGFR stimulated by Ras‐mediated MAPK activation.^48^ It is taken for granted that EGFR‐targeted therapy is theoretically a promising anti‐GBM therapy. However, the clinical efficacy of this therapy has been only modest in GBM patients.^49^, ^50^ Therefore, a better understanding in the mechanism of EGFR activation might provide clues for EGFR‐targeted GBM treatment.

In this study, we found that expression of EGFR and its downstream effectors Akt and Erk1/2 were positively correlated with MYST1 expression in a TGGA clinical cohort (N = 538, Figure 5C‐E). Besides, MYST1 silencing inactivated EGFR signaling pathways while MYST1 overexpression activated EGFR signaling both in vitro and in vivo (Figures 5F,G, 6A,F). These results predicted the potential role of MYST1 in GBM. KM analysis showed that high MYST1 expression was significantly correlated with poorer prognosis in patients with glioma and GBM (Figure 1F‐I). Besides, MYST1 silencing in several GBM cell lines significantly impedes cell proliferation while MYST1 overexpression promotes cell proliferation in vitro (Figure 2C‐E, 6C). MYST1 downregulation or upregulation also played important roles in tumorigenecity in nude mice (Figures 4, 6D,E). All these data showed that MYST1 played important role in the development of GBM.

Erlotinib (Tarceva), a specific inhibitor of EGFR, has approved by the US Food and Drug Administration (FDA) for the treatment of NSCLC. Erlotinib in combination with chemotherapy has improved OS in patients with NSCLC by 19%, and has improved progression‐free survival by 29%, when compared to chemotherapy alone.^51^ In addition, the FDA has approved erlotinib in combination with gemcitabine for the treatment of pancreatic cancer in November 2005. In lung cancer, erlotinib has been shown to be effective in patients with or without EGFR mutations, but appears to be more effective in patients with EGFR mutations.^52^, ^53^ However, erlotinib alone or in combination with conventional adjuvant therapies has not shown to represent a major success for GBM treatment.^54^ Besides, Try1068‐phosphrylated EGFR is also shown to be a target of erlotinib.^55^ In this study, we used erlotinib to block EGFR and the result showed that MYST1 overexpression‐promoted cell proliferation and EGFR activation were rescued after EGFR blockade (Figure 7). These results showed that MYST1 promoted tumor progression via activation of EGFR in GBM. The understanding of function of MYST1 might provide clues for EGFR‐target therapy in GBM. However, EGFR mRNA expression remained unchanged after MYST1 silence or overexpression (Figures 5H, 6B). Then we showed that MYST1 might epigenetically control the transcription of EGFR ligand EGF (Figure 8). These results mean that MYST1 controls the EGFR signalling probably though promoting the expression of EGF.

In addition, we analyzed the genes related to MYST1 expression in a cohort of glioma and the mini ontology analysis revealed that cell cycle was the most correlated biological process that MYST1 might involve (Figure 3A). Furthermore, we found that MYST1 silencing induced cell cycle arrest at G2/M phase as well as decrease in cell cycle‐related proteins such as CDK1, Cyclin A, and Cyclin B1, and upregulation of p21 in GBM cells (Figure 3B‐D). In consistent with our reports, in androgen receptor‐transformed PC3 prostate cancer, MYST1 depletion also induces p21 upregulation, which results in G2M arrest.^56^ Other study also showed that MYST1/MOF directly binds and maintains the expression of genes required for cell cycle progression in proliferating cells.^15^ Besides, in the mini ontology analysis, DNA repair, which might be a reason for G2M phase arrest, was the third most related biological process (Figure 3A). Many studies also showed that MYST1/MOF played pivotal roles in responding to DNA damage.^16^, ^57^, ^58^ In addition, It was reported that EGFR‐AKT/ERK signaling pathway was a major regulator of G2M phase in GBM.^59^ Downregulated EGFR‐AKT/ERK signaling pathway by MYST1 silencing might also be a reason for G2/M phase arrest. However, much more work should be done to further explain the function of MYST1 in GBM.

In summary, we reported that MYST1 expression was negatively correlated with 3‐ or 5‐year survival rate in patients with glioma. Our results demonstrated that MYST1 silencing in GBM cells impeded cell proliferation and cell cycle procession in vitro, as well as inhibited tumor formation in in vivo. Besides, MYST1 overexpression promoted tumor progression in vitro and in vivo. Importantly, EGFR signaling pathway was activated by MYST1 while inhibited by MYST1 silencing. EGFR inhibitor erlotinib recovered MYST1 overexpression‐induced cell proliferation. MYST1 controlled EGFR signaling possibly through epigenetically promoting the expression of EGFR ligand, EGF. Our data showed the pivotal roles of MYST1 in the regulation of tumor progression in GBM and might provide clues for GBM treatment.

## CONFLICT OF INTEREST

The authors have no conflict of interest.

## Data Availability

The data that support the findings of this study are available from the corresponding author upon reasonable request.

## References

[1] Preusser M , de Ribaupierre S , Wöhrer A , et al. Current concepts and management of glioblastoma. Ann Neurol. 2011;70(1):9‐21. 21786296 10.1002/ana.22425

[2] Weller M , Cloughesy T , Perry JR , Wick W . Standards of care for treatment of recurrent glioblastoma–are we there yet? Neuro Oncol. 2013;15(1):4‐27. 23136223 10.1093/neuonc/nos273 PMC3534423

[3] Stupp R , Mason WP , van den Bent MJ , et al. Radiotherapy plus concomitant and adjuvant temozolomide for glioblastoma. N Engl J Med. 2005;352(10):987‐996. 15758009 10.1056/NEJMoa043330

[4] Stupp R , Hegi ME , Mason WP , et al. Effects of radiotherapy with concomitant and adjuvant temozolomide versus radiotherapy alone on survival in glioblastoma in a randomised phase III study: 5‐year analysis of the EORTC‐NCIC trial. Lancet Oncol. 2009;10(5):459‐466. 19269895 10.1016/S1470-2045(09)70025-7

[5] Rosenquist R , Esteller M , Plass C . Introduction: epigenetics in cancer. Semin Cancer Biol. 2018;51:iv‐v. 29990621 10.1016/j.semcancer.2018.07.002

[6] Gusyatiner O , Hegi ME . Glioma epigenetics: from subclassification to novel treatment options. Semin Cancer Biol. 2018;51:50‐58. 29170066 10.1016/j.semcancer.2017.11.010

[7] Dong Z , Cui H . Epigenetic modulation of metabolism in glioblastoma. Semin Cancer Biol. 2019;57:45‐51. 30205139 10.1016/j.semcancer.2018.09.002

[8] Mellert HS , McMahon SB . hMOF, a KAT(8) with many lives. Mol Cell. 2009;36(2):174‐175. 19854127 10.1016/j.molcel.2009.10.005

[9] Yuan H , Rossetto D , Mellert H , et al. MYST protein acetyltransferase activity requires active site lysine autoacetylation. EMBO J. 2012;31(1):58‐70. 22020126 10.1038/emboj.2011.382 PMC3252582

[10] Su J , Wang F , Cai Y , Jin J . The functional analysis of histone acetyltransferase MOF in tumorigenesis. Int J Mol Sci. 2016;17:99. 10.3390/ijms17010099 PMC4730341 26784169

[11] Sykes SM , Mellert HS , Holbert MA , et al. Acetylation of the p53 DNA‐binding domain regulates apoptosis induction. Mol Cell. 2006;24(6):841‐851. 17189187 10.1016/j.molcel.2006.11.026 PMC1766330

[12] Sharma GG , So S , Gupta A , et al. MOF and histone H4 acetylation at lysine 16 are critical for DNA damage response and double‐strand break repair. Mol Cell Biol. 2010;30(14):3582‐3595. 20479123 10.1128/MCB.01476-09 PMC2897562

[13] Smith ER , Cayrou C , Huang R , Lane WS , Cote J , Lucchesi JC . A human protein complex homologous to the Drosophila MSL complex is responsible for the majority of histone H4 acetylation at lysine 16. Mol Cell Biol. 2005;25(21):9175‐9188. 16227571 10.1128/MCB.25.21.9175-9188.2005 PMC1265810

[14] Gupta A , Guerin‐Peyrou TG , Sharma GG , et al. The mammalian ortholog of Drosophila MOF that acetylates histone H4 lysine 16 is essential for embryogenesis and oncogenesis. Mol Cell Biol. 2008;28(1):397‐409. 17967868 10.1128/MCB.01045-07 PMC2223300

[15] Sheikh BN , Bechtel‐Walz W , Lucci J , et al. MOF maintains transcriptional programs regulating cellular stress response. Oncogene. 2016;35(21):2698‐2710. 26387537 10.1038/onc.2015.335 PMC4893634

[16] Gupta A , Hunt C , Hegde M , et al. MOF phosphorylation by ATM regulates 53BP1‐mediated double‐strand break repair pathway choice. Cell Rep. 2014;8(1):177‐189. 24953651 10.1016/j.celrep.2014.05.044 PMC4300955

[17] Pfister S , Rea S , Taipale M , et al. The histone acetyltransferase hMOF is frequently downregulated in primary breast carcinoma and medulloblastoma and constitutes a biomarker for clinical outcome in medulloblastoma. Int J Cancer. 2008;122(6):1207‐1213. 18058815 10.1002/ijc.23283

[18] Cai M , Hu Z , Liu J , et al. Expression of hMOF in different ovarian tissues and its effects on ovarian cancer prognosis. Oncol Rep. 2015;33(2):685‐692. 25483274 10.3892/or.2014.3649

[19] Cao L , Zhu L , Yang J , et al. Correlation of low expression of hMOF with clinicopathological features of colorectal carcinoma, gastric cancer and renal cell carcinoma. Int J Oncol. 2014;44(4):1207‐1214. 24452485 10.3892/ijo.2014.2266

[20] Wang Y , Zhang R , Wu D , et al. Epigenetic change in kidney tumor: downregulation of histone acetyltransferase MYST1 in human renal cell carcinoma. J Exp Clin Cancer Res. 2013;32:8. 23394073 10.1186/1756-9966-32-8 PMC3577515

[21] Chen Z , Ye X , Tang N , et al. The histone acetylranseferase hMOF acetylates Nrf2 and regulates anti‐drug responses in human non‐small cell lung cancer. Br J Pharmacol. 2014;171(13):3196‐3211. 24571482 10.1111/bph.12661 PMC4080974

[22] Luo H , Shenoy A , Li X , et al. MOF acetylates the histone demethylase LSD1 to suppress epithelial‐to‐mesenchymal transition. Cell Rep. 2016;15(12):2665‐2678. 27292636 10.1016/j.celrep.2016.05.050

[23] Wang M , Liu Y , Zou J , et al. Transcriptional co‐activator TAZ sustains proliferation and tumorigenicity of neuroblastoma by targeting CTGF and PDGF‐beta. Oncotarget. 2015;6(11):9517‐9530. 25940705 10.18632/oncotarget.3367 PMC4496235

[24] Dong Z , Lei Q , Yang R , et al. Inhibition of neurotensin receptor 1 induces intrinsic apoptosis via let‐7a‐3p/Bcl‐w axis in glioblastoma. Br J Cancer. 2017;116(12):1572‐1584. 28494471 10.1038/bjc.2017.126 PMC5518855

[25] Rot S , Taubert H , Bache M , et al. Low HIF‐1α and low EGFR mRNA expression significantly associate with poor survival in soft tissue sarcoma patients; the proteins react differently. Int J Mol Sci. 2018;19(12):3842. 10.3390/ijms19123842 PMC6321736 30513863

[26] Liu L , Dong Z , Lei Q , et al. Inactivation/deficiency of DHODH induces cell cycle arrest and programed cell death in melanoma. Oncotarget. 2017;8(68):112354‐112370. 29348830 10.18632/oncotarget.19379 PMC5762515

[27] Yang R , Yi L , Dong Z , et al. Tigecycline inhibits glioma growth by regulating miRNA‐199b‐5p‐HES1‐AKT pathway. Mol Cancer Ther. 2016;15(3):421‐429. 26823491 10.1158/1535-7163.MCT-15-0709

[28] Zhao Y , He J , Li J , et al. Demethylzeylasteral inhibits cell proliferation and induces apoptosis through suppressing MCL1 in melanoma cells. Cell Death Dis. 2017;8(10):e3133. 29072681 10.1038/cddis.2017.529 PMC5682691

[29] Tang Z , Li C , Kang B , Gao G , Li C , Zhang Z . GEPIA: a web server for cancer and normal gene expression profiling and interactive analyses. Nucleic Acids Res. 2017;45(W1):W98‐w102. 28407145 10.1093/nar/gkx247 PMC5570223

[30] Bowman RL , Wang Q , Carro A , Verhaak RG , Squatrito M . GlioVis data portal for visualization and analysis of brain tumor expression datasets. Neuro Oncol. 2017;19(1):139‐141. 28031383 10.1093/neuonc/now247 PMC5193031

[31] Rai TS , Cole JJ , Nelson DM , et al. HIRA orchestrates a dynamic chromatin landscape in senescence and is required for suppression of neoplasia. Genes Dev. 2014;28(24):2712‐2725. 25512559 10.1101/gad.247528.114 PMC4265675

[32] Robinson JT , Thorvaldsdóttir H , Winckler W , et al. Integrative genomics viewer. Nat Biotechnol. 2011;29(1):24‐26. 21221095 10.1038/nbt.1754 PMC3346182

[33] Thorvaldsdóttir H , Robinson JT , Mesirov JP . Integrative Genomics Viewer (IGV): high‐performance genomics data visualization and exploration. Briefings Bioinf. 2012;14(2):178‐192. 10.1093/bib/bbs017 PMC3603213 22517427

[34] Robinson JT , Thorvaldsdóttir H , Wenger AM , Zehir A , Mesirov JP . Variant review with the integrative genomics viewer. Cancer Res. 2017;77(21):e31‐e34. 29092934 10.1158/0008-5472.CAN-17-0337 PMC5678989

[35] Gleize V , Alentorn A , Connen de Kerillis L , et al. CIC inactivating mutations identify aggressive subset of 1p19q codeleted gliomas. Ann Neurol. 2015;78(3):355‐374. 26017892 10.1002/ana.24443

[36] Bettegowda C , Agrawal N , Jiao Y , et al. Mutations in *CIC* and *FUBP1* contribute to human oligodendroglioma. Science. 2011;333(6048):1453‐1455. 21817013 10.1126/science.1210557 PMC3170506

[37] Cancer Genome Atlas Research Network . Comprehensive genomic characterization defines human glioblastoma genes and core pathways. Nature. 2008;455(7216):1061‐1068. 18772890 10.1038/nature07385 PMC2671642

[38] Brennan C , Verhaak R , McKenna A , et al. The somatic genomic landscape of glioblastoma. Cell. 2013;155(2):462‐477. 24120142 10.1016/j.cell.2013.09.034 PMC3910500

[39] Parsons DW , Jones S , Zhang X , et al. An integrated genomic analysis of human glioblastoma multiforme. Science. 2008;321(5897):1807‐1812. 18772396 10.1126/science.1164382 PMC2820389

[40] Verhaak R , Hoadley KA , Purdom E , et al. Integrated genomic analysis identifies clinically relevant subtypes of glioblastoma characterized by abnormalities in PDGFRA, IDH1, EGFR, and NF1. Cancer Cell. 2010;17(1):98‐110. 20129251 10.1016/j.ccr.2009.12.020 PMC2818769

[41] Dunn GP , Rinne ML , Wykosky J , et al. Emerging insights into the molecular and cellular basis of glioblastoma. Genes Dev. 2012;26(8):756‐784. 22508724 10.1101/gad.187922.112 PMC3337451

[42] Nishikawa R , Sugiyama T , Narita Y , Furnari F , Cavenee WK , Matsutani M . Immunohistochemical analysis of the mutant epidermal growth factor, deltaEGFR, in glioblastoma. Brain Tumor Pathol. 2004;21(2):53‐56. 15700833 10.1007/BF02484510

[43] Nadeem Abbas M , Kausar S , Wang F , Zhao Y , Cui H . Advances in targeting the epidermal growth factor receptor pathway by synthetic products and its regulation by epigenetic modulators as a therapy for glioblastoma. Cells. 2019;8(4):350. 10.3390/cells8040350 PMC6523687 31013819

[44] Roth P , Weller M . Challenges to targeting epidermal growth factor receptor in glioblastoma: escape mechanisms and combinatorial treatment strategies. Neuro Oncol. 2014;16(suppl 8):viii14‐viii19. 25342600 10.1093/neuonc/nou222 PMC4207136

[45] Thorne AH , Zanca C , Furnari F . Epidermal growth factor receptor targeting and challenges in glioblastoma. Neuro Oncol. 2016;18(7):914‐918. 26755074 10.1093/neuonc/nov319 PMC4896544

[46] Bazley LA , Gullick WJ . The epidermal growth factor receptor family. Endocr Relat Cancer. 2005;12(Suppl 1):S17‐S27. 16113093 10.1677/erc.1.01032

[47] Schlessinger J . Common and distinct elements in cellular signaling via EGF and FGF receptors. Science. 2004;306(5701):1506‐1507. 15567848 10.1126/science.1105396

[48] Rojas M , Yao S , Lin YZ . Controlling epidermal growth factor (EGF)‐stimulated Ras activation in intact cells by a cell‐permeable peptide mimicking phosphorylated EGF receptor. J Biol Chem. 1996;271(44):27456‐27461. 8910327 10.1074/jbc.271.44.27456

[49] Schulte A , Liffers K , Kathagen A , et al. Erlotinib resistance in EGFR‐amplified glioblastoma cells is associated with upregulation of EGFRvIII and PI3Kp110delta. Neuro Oncol. 2013;15(10):1289‐1301. 23877316 10.1093/neuonc/not093 PMC3779041

[50] Rich JN , Reardon DA , Peery T , et al. Phase II trial of gefitinib in recurrent glioblastoma. J Clin Oncol. 2004;22(1):133‐142. 14638850 10.1200/JCO.2004.08.110

[51] Cappuzzo F , Ciuleanu T , Stelmakh L , et al. SATURN: A double‐blind, randomized, phase III study of maintenance erlotinib versus placebo following nonprogression with first‐line platinum‐based chemotherapy in patients with advanced NSCLC. J Clin Oncol. 2009;27(15_suppl):8001.

[52] Kobayashi K , Hagiwara K . Epidermal growth factor receptor (EGFR) mutation and personalized therapy in advanced nonsmall cell lung cancer (NSCLC). Targeted Oncol. 2013;8(1):27‐33. 10.1007/s11523-013-0258-9 PMC3591525 23361373

[53] Qi W‐X , Shen Z , Lin F , et al. Comparison of the efficacy and safety of EFGR tyrosine kinase inhibitor monotherapy with standard second‐line chemotherapy in previously treated advanced non‐small‐cell lung cancer: a systematic review and meta‐analysis. Asian Pac J Cancer Prev. 2012;13(10):5177‐5182. 23244131 10.7314/apjcp.2012.13.10.5177

[54] Karpel‐Massler G , Westhoff MA , Kast RE , Wirtz CR , Halatsch ME . Erlotinib in glioblastoma: lost in translation? Anticancer Agents Med Chem. 2011;11(8):748‐755. 21707495 10.2174/187152011797378788

[55] Sette G , Salvati V , Mottolese M , et al. Tyr1068‐phosphorylated epidermal growth factor receptor (EGFR) predicts cancer stem cell targeting by erlotinib in preclinical models of wild‐type EGFR lung cancer. Cell Death Dis. 2015;6(8):e1850. 26247735 10.1038/cddis.2015.217 PMC4558509

[56] Jaganathan A , Chaurasia P , Xiao GQ , et al. Coactivator MYST1 regulates nuclear factor‐kappaB and androgen receptor functions during proliferation of prostate cancer cells. Mol Endocrinol. 2014;28(6):872‐885. 24702180 10.1210/me.2014-1055 PMC4042067

[57] Li X , Corsa C , Pan PW , et al. MOF and H4 K16 acetylation play important roles in DNA damage repair by modulating recruitment of DNA damage repair protein Mdc1. Mol Cell Biol. 2010;30(22):5335‐5347. 20837706 10.1128/MCB.00350-10 PMC2976376

[58] Gupta A , Sharma GG , Young C , et al. Involvement of human MOF in ATM function. Mol Cell Biol. 2005;25(12):5292‐5305. 15923642 10.1128/MCB.25.12.5292-5305.2005 PMC1140595

[59] Fan QW , Weiss WA . RNA interference against a glioma‐derived allele of EGFR induces blockade at G2M. Oncogene. 2005;24(5):829‐837. 15580296 10.1038/sj.onc.1208227

